# The anti-angiogenic tyrosine kinase inhibitor Pazopanib kills cancer cells and disrupts endothelial networks in biomimetic three-dimensional renal tumouroids

**DOI:** 10.1177/2041731420920597

**Published:** 2020-05-18

**Authors:** Katerina Stamati, Patricia A Redondo, Agata Nyga, Joana B Neves, Maxine GB Tran, Mark Emberton, Umber Cheema, Marilena Loizidou

**Affiliations:** 1 Research Department of Surgical Biotechnology, Division of Surgery & Interventional Science, University College London, London, UK; 2 Specialist Centre for Kidney Cancer, Royal Free London NHS Foundation Trust, London, UK; 3 Research Department of Targeted Intervention, Division of Surgery & Interventional Science, University College London, London, UK; 4 Department of Urology, University College London Hospitals NHS Foundation Trust, London, UK

**Keywords:** 2D versus 3D culture, in vitro endothelial networks, Pazopanib, renal cell carcinoma, tumouroid

## Abstract

Pazopanib is a tyrosine kinase inhibitor used to treat renal cell carcinoma. Few in vitro studies investigate its effects towards cancer cells or endothelial cells in the presence of cancer. We tested the effect of Pazopanib on renal cell carcinoma cells (CAKI-2,786-O) in two-dimensional and three-dimensional tumouroids made of dense extracellular matrix, treated in normoxia and hypoxia. Finally, we engineered complex tumouroids with a stromal compartment containing fibroblasts and endothelial cells. Simple CAKI-2 tumouroids were more resistant to Pazopanib than 786-O tumouroids. Under hypoxia, while the more ‘resistant’ CAKI-2 tumouroids showed no decrease in viability, 786-O tumouroids required higher Pazopanib concentrations to induce cell death. In complex tumouroids, Pazopanib exposure led to a reduction in the overall cell viability (p < 0.0001), disruption of endothelial networks and direct killing of renal cell carcinoma cells. We report a biomimetic multicellular tumouroid for drug testing, suitable for agents whose primary target is not confined to cancer cells.

## Introduction

Renal cell carcinoma (RCC) is classified histologically into clear cell (75%–80%), papillary (7%–14%) and chromophobe (6–11%).^1^ Patients with advanced RCC and a favourable prognostic risk profile are treated in first line with Sunitinib or Pazopanib.^2^ These drugs may also be a first-line option for patients who are unsuitable for checkpoint inhibitors or can be used in second-/third-line treatment scenarios. Pazopanib, a tyrosine kinase inhibitor (TKI),^1,3^ inhibits receptors which drive angiogenesis and cell viability, for example, vascular endothelial growth factor receptors VEGFR1, 2, 3; platelet-derived growth factor receptors PDGFR-α, PDGFRβ; and c-KIT.^3–5^ Sunitinib has a similar mechanism of action as Pazopanib with the two drugs showing similar efficacy in patients, with one study showing that Pazopanib was favoured for better safety and quality-of-life outcomes.^6^ Evaluating TKI action using in vitro cancer models is challenging as their main target is endothelial cells via VEGFR abrogation. However, their biochemical profile suggests they may act directly against RCC cells. There have been few studies studying TKI effects on RCC cells in vitro, and these report contradictory results.^7–9^

Several groups have been developing in vitro cellular models, moving from two-dimensional (2D) culture towards more biomimetic three-dimensional (3D) systems,^10,11^ using mainly immortalised cell lines,^12,13^ as research using patient-derived cells remains challenging.^14–19^ Recently, some 3D models have reflected the growing appreciation of the biophysical microenvironment in which cancers reside.^20,21^ We also developed a 3D dense tissue mimetic using collagen type I, named tumouroid, to enable us to mimic biophysical cues.^22–27^ Recapitulating elements of this microenvironment is critical if we are to see a meaningful response to drug interventions.

Here, we investigated how RCC lines respond to Pazopanib using in vitro models of different complexity, aiming to set parameters before transitioning to using patient-derived cells.^28^ Due to the similarities between the two main TKIs, we chose to use Pazopanib in our study. We used two clear cell RCC (ccRCC) lines, 786-O which harbours a mutation in von Hippel-Lindau (VHL) and CAKI-2 which expresses wild-type VHL, as this is the most common mutation in patients with ccRCC.^29^ We tested drug efficacy in 2D monocultures and tumouroids where we focused on engineering three elements of the biophysical cancer niche. First, we cultured cancer cells in dense, tissue-like extracellular matrices (ECMs); second, we established tumouroids in hypoxia (1% O_2_) to mimic tissue conditions; third, we engineered a distinct stromal compartment which the cancer mass interacts with. This allowed us to demonstrate for the first time the response to Pazopanib of both cancer and endothelial cellular structures within the surrounding stroma in an in vitro RCC model.

## Methods

### Cells

CAKI-2 and 786-O RCC cell lines (European Collection of Authenticated Cell Cultures, Public Health England, UK), both representative of ccRCC, were cultured in RPMI medium supplemented with 10% foetal bovine serum (FBS) (Gibco, UK) and 1% penicillin–streptomycin (P/S, 10,000 units of penicillin and 10 mg of streptomycin/mL; Sigma, UK). Human umbilical vein endothelial cells (HUVECs; PromoCell, Germany) were cultured in Endothelial Growth Medium supplemented with 1% P/S and 5% FBS and used up to passage 5. Human dermal fibroblasts (HDFs; Lonza, UK) were grown in Dulbecco’s Modified Eagle Medium (DMEM; 4.5 g/L glucose) with 10% FBS and 1% P/S. Cells were routinely maintained as 2D monocultures in a humidified atmosphere at 37°C, 20% O_2_, 5%CO_2_.

### Tumouroid manufacture

**Figure 1.**
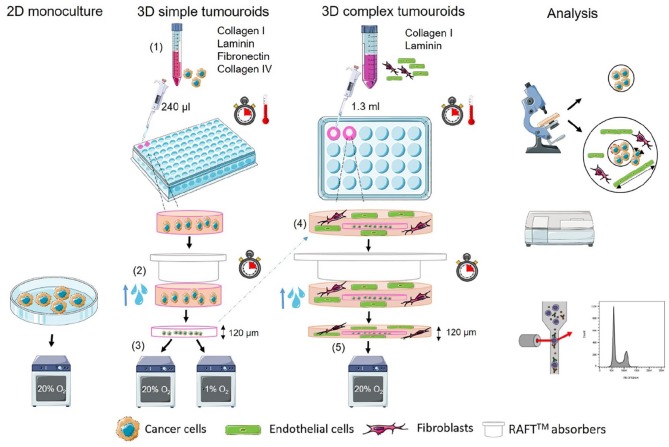
Schematic of tumouroid manufacture. (1) Cancer cell lines are embedded in collagen I hydrogels in 96-well plates containing collagen IV, laminin and fibronectin. (2) Interstitial fluid is removed using commercial absorbers (RAFT^TM^) to create a dense cancer mass. (3) The resulting cancer mass–only tumouroid is cultured and treated with drugs. (4) For complex tumouroid manufacture, the cancer-only simple tumouroid is nested in another hydrogel containing human dermal fibroblasts (HDFs) and human umbilical vein endothelial cells (HUVECs) in 24-well plates and compressed using commercial absorbers (RAFTTM) to create a dense complex tumouroid. (5) Complex tumouroids are cultured and treated with drugs. Analysis is done using fluorescence imaging, cell viability, ELISA and cell cycle analysis using flow cytometry. Schematic is based on previous works^23,26,27^ and designed using SMART – Servier Medical ART.

(1) Simple tumouroids were manufactured as previously described.^23,26^ Rat tail collagen type I (2.05 mg/mL; First Link, UK, 80% of final volume), 10× Minimal Essential Medium (MEM) (Gibco; 10% of final volume) and neutralised solution (6% of final volume) were mixed, before adding cells in the media (4% of final volume). Collagen IV, laminin (VWR, UK) and fibronectin (Millipore, UK) were added at 5 µg/mL. Solutions were kept on ice. Hydrogels were allowed to set at 37°C for 15 min before using hydrophilic RAFT absorbers (Lonza) to remove excess fluid for a further 15 min. A total of 240 µL of cell collagen mixture containing 20,000 cancer cells was used per well in a 96-well plate, in fully supplemented RPMI medium.
(2) Complex tumouroids comprised two compartments: cancer cell–containing simple tumouroids were embedded within stromal compartments containing HUVECs (100,000) and HDFs (25,000) in collagen (1.3 mL) with laminin (50 µg/mL).^23,26,30^ Absorbers were used as above and tumouroids cultured (10 days) in mixed media (1:1:1) before drug testing (Figure 1). Cell ratios have been previously reported.^26^

### Pazopanib

Pazopanib hydrochloride was prepared in dimethyl sulfoxide (DMSO) at 35.86 mM, stored at −20°C (Generon, UK) and further diluted with culture media. Media-only and DMSO vehicle controls were used throughout. Different treatment protocols were optimised using 786-O cells. For some specific treatments, normoxic (20% O_2_) and hypoxic (1% O_2_) culture conditions were compared.

### Immunofluorescence

Tumouroids were fixed for 30 min, at room temperature, in 10% neutral buffered formalin and washed 3 times in phosphate-buffered saline (PBS) for 10 min. Following permeabilisation with 0.3% Triton X (Sigma) and blocking in 1% bovine serum albumin, tumouroids were stained using phalloidin 488 (1:200; Invitrogen, UK), mouse anti-human CD31 and/or vimentin 594 (1:200, ab9498; 1:1000, ab154207; Abcam, UK). For CD31 staining, we used a chicken anti-mouse Alexa Fluor 488 secondary antibody (1:500; Invitrogen). DAPI mounting medium was used to counterstain cell nuclei (Vector Labs; Vectashield). Image J (National Institutes of Health (NIH), USA) software was used for analysis of spheroid sizes and cell invasion. Spheroid sizes were calculated by outlining spheroids using Image J software and measuring the surface area. Cell invasion in complex tumouroids was measured as the distance travelled from the cancer mass boundary into the surrounding stroma, which can be easily identified both macroscopically and microscopically (Figure 1, analysis).

### Viability assay

CellTiter Glo 3D (Promega, UK) determines ATP levels. Equal volumes of culture supernatant and CellTiter Glo (2D: 100 µL, simple tumouroids: 50 µl, complex tumouroids: 250 µL) were mixed vigorously for 30 s using a Tecan plate reader at maximum speed. The plate was incubated, at room temperature for 25 min, and luminescence was measured (Tecan, UK).

### Cell cycle analysis

To ensure sufficient cell numbers, five tumouroids were pooled and digested (collagenase type I, 37°C, 1 h) on a shaker (200 IU/mL in Hanks’ Balanced Salt Solution (HBSS)/calcium/magnesium; Gibco). The digest was centrifuged at 700*g* for 5 min. The supernatant was discarded, and cells were incubated in TrypLE Express (Gibco) to obtain a single cell suspension, washed by centrifugation and resuspended in 250 µL PBS. Ice cold 70% ethanol was added dropwise (2 mL) to the cell pellet while vortexing and cells stored in the fridge, overnight. For processing, cells were centrifuged twice at 800*g* for 5 min, stained using FxCycle PI (Invitrogen) for 20–30 min at room temperature, and flow cytometry analysis performed on a BD LSRII using FACS DIVA and FlowJo software.

### ELISA

Vascular endothelial growth factor (VEGF) can be overproduced by cancer cells in response to activation of the hypoxia pathway. Reduced VEGF production can signal cell inactivity and/or death. Supernatants were removed at the culture end point and stored at −80°C until needed. Samples were diluted prior to use: 40× for 786-O and 4× for CAKI-2. VEGF Quantikine ELISA kits were used as per the manufacturer’s instructions (Bio-Techne, UK). Results were measured using absorbance at 450 nm and correction at 540 nm and compared to the standards provided.

### Statistical analysis

Results are shown as mean ± SD. Statistical significance was calculated using GraphPad Prism. We used one-way analysis of variance (ANOVA) with Tukey post hoc analysis (parametric), or Kruskal–Wallis with Dunn’s post hoc analysis (non-parametric), as appropriate. Mann–Whitney U test was used when only two groups of samples were compared (non-parametric). A typical experiment consisted of three independent repeats, with triplicate points in each independent repeat. Significance was set at p < 0.05, and p values are shown as *p < 0.05, **p < 0.01, ***p < 0.001 and ****p < 0.0001.

## Results

### RCC cells respond to Pazopanib in 2D culture

**Figure 2.**
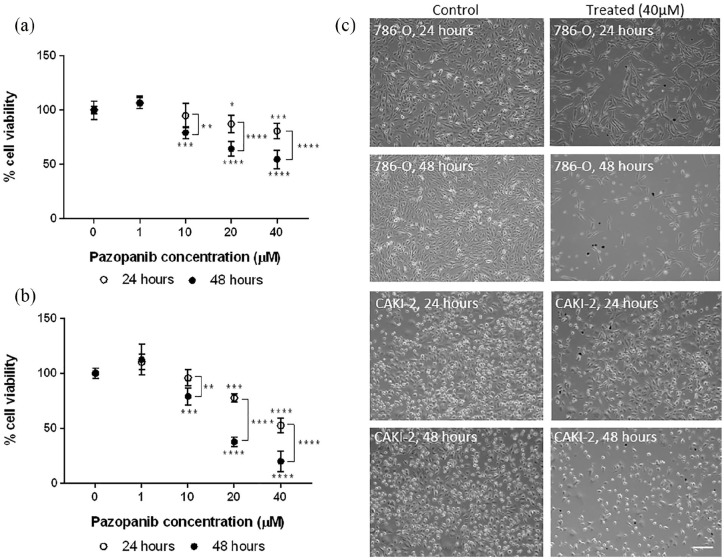
Renal cell carcinoma cell lines treated with Pazopanib in 2D culture. (a) Cell viability (CellTiter Glo) of 786-O cell line treated for 24 and 48 h with Pazopanib. (b) Cell viability of CAKI-2 cell line treated for 24 and 48 h with Pazopanib. (c) Representative images of 786-O (upper four) and CAKI-2 (lower four) cells, control cells (0 µM Pazopanib) on the left and treated with 40 µM Pazopanib on the right. Scale bar = 200 µm. Two-way ANOVA with Tukey’s post hoc analysis. Asterisks above open circles indicate significance to control at 24 h of treatment and asterisks below dark circles indicate significance to control cells at 48 h. *p < 0.05; **p < 0.01; ***p < 0.001; ****p < 0.0001.

We tested Pazopanib response in 2D, after 24- and 48-h treatment (Figure 2(a) and (b)). Cells showed a dose-dependent significant decrease in viability (786-O, p < 0.0001; CAKI-2, p < 0.001), with 786-O cells displaying higher sensitivity to Pazopanib, demonstrated by CellTiter Glo and imaging (Figure 2(c)). Increasing exposure time to 48 h had a significant effect, with viability in 786-O cells decreasing from 52% (24 h) to 20% (48 h) and in CAKI-2 from 80% to 54%, at 40 µM (p < 0.0001).

### Mature tumouroids are less responsive to Pazopanib compared to early tumouroids

**Figure 3.**
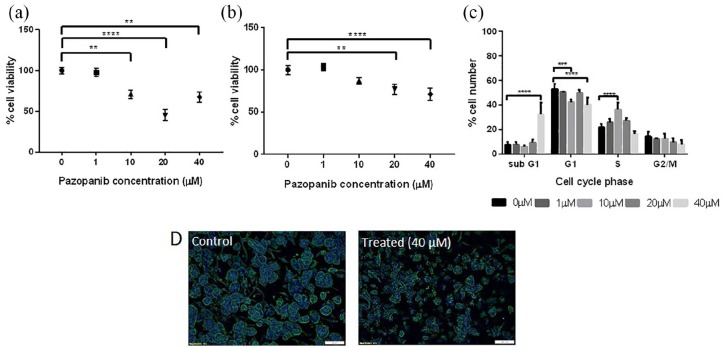
Renal tumouroids, manufactured with 786-O cells, were treated with Pazopanib for 72 h either on day 1 (early) or on day 10 after manufacture (mature). Cell viability (CellTiter Glo) in (a) early tumouroids and (b) mature tumouroids. (c) A shift into subG1 of cancer cells treated at high concentrations seen in mature tumouroids. (d) Fluorescent images of cancer spheroids within mature tumouroids for control (left panel, control 0 µM Pazopanib) and treated samples (right panel, 40 µM Pazopanib), demonstrating effect on spheroid size and morphology (Phalloidin staining, DAPI counterstain). ***p < 0.001; **** p < 0.0001, Kruskal–Wallis with Dunn’s post hoc analysis. Scale bar = 100 µm.

We used 786-O simple tumouroids to optimise treatment protocols, as the cells showed greater response in 2D. ‘Early’ tumouroids, at day 1 post-manufacture, were exposed to Pazopanib for 72 h and showed a significant reduction in viability for 10, 20 and 40 µM (71%, 45% and 67% viability, respectively; p < 0.001), as measured by CellTiter Glo (Figure 3(a)). It should be noted that the viability increase seen between 20 and 40 µM was not significant, although there was a trend towards increased viability at the higher drug concentration. This was also found in other tumouroid conditions tested and fit the hormetic dose–response model. This model is commonly reported and is characterised by low-dose stimulation and high-dose inhibition.^31,32^

However, tumouroids treated at day 1 contain mostly single cells (Supplementary Figure 1). In order to treat cells at a more in vivo–like state, tumouroids were cultured for 10 days to allow cancer cells to form spheroids/aggregates (mature tumouroids) prior to drug treatment (Supplementary Figure 1). After 72 h of exposure, response to Pazopanib was observable but less pronounced than that of early tumouroids (Figure 3(b)). There was no significant response to 10 µM and a maximum of 30% cell death with 40 µM Pazopanib (Figure 3(b)). Cell cycle analysis showed an increase in subG1 events only at 40 µM (p < 0.0001, Figure 3(c)), with fewer, smaller spheroids (qualitative imaging observation, Figure 3(d)).

### Hypoxia decreases response to Pazopanib in 786-O mature tumouroids

**Figure 4.**
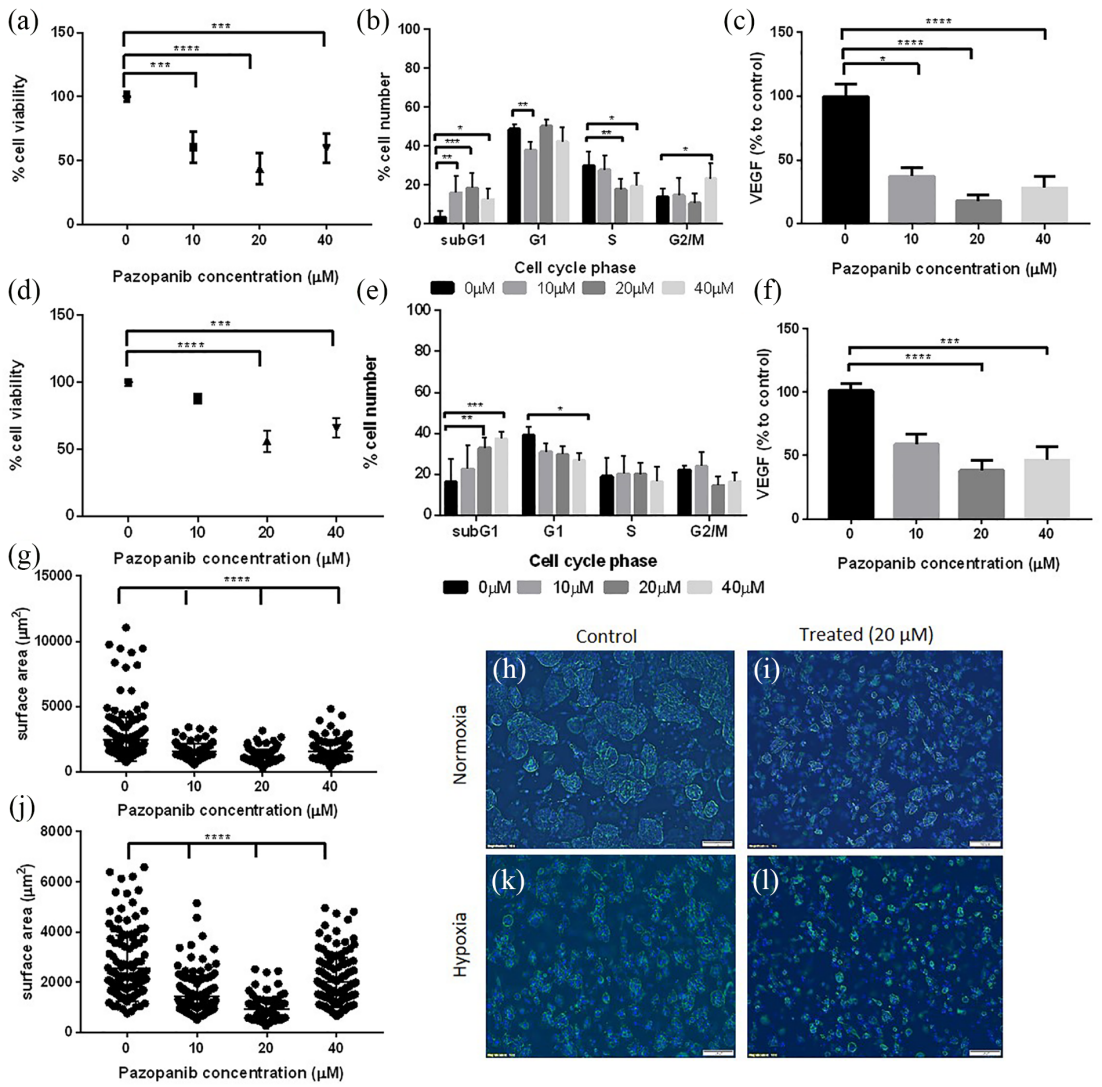
Mature tumouroids manufactured using 786-O cells, treated with Pazopanib. Tumouroids were exposed to Pazopanib at day 10 of manufacture, in normoxia or hypoxia, for a total of 120 h. Normoxia (20% O_2_): a, b, c, g, h, i; hypoxia (1% O_2_): d, e, f, j, k, l. (a) Cell viability measured using CellTiter Glo after treatment with Pazopanib (normoxic conditions). (b) Cell cycle analysis of treated tumouroids (normoxic conditions). (c) VEGF levels in treated tumouroids measured using ELISA, shown as a percentage to untreated tumouroids (normoxic conditions). (d) Cell viability measured using CellTiter Glo after treatment with Pazopanib in hypoxia (1% O_2_). (e) Cell cycle analysis of treated tumouroids (hypoxic conditions). (f) VEGF levels in the supernatant of treated tumouroids shown as a percentage to untreated tumouroid (ELISA, hypoxic conditions). (g, j) Surface area of spheroids following treatment in (g) normoxia or (j) hypoxia demonstrating effect of treatment on spheroid size. (h, i, k, l) Fluorescent images of cancer spheroids within mature tumouroids for (h, k) control and (i, l) treated tumouroids in (h, i) normoxia or (k, l) hypoxia (Phalloidin staining, DAPI counterstain; scale bar = 100 µm). **p < 0.01; ****p < 0.0001, Kruskal–Wallis with Dunn’s post hoc analysis.

To maximise drug effectiveness, we administered Pazopanib to mature 786-O tumouroids for 120 h, replenishing with drug at 72 h. We observed an increase in cell death, with a significant reduction in viability at 10, 20 and 40 µM Pazopanib (60%, p < 0.001; 43%, p < 0.0001; 59%, p < 0.001 viability, respectively; Figure 4(a)). Cell cycle analysis revealed a significant increase in subG1 events at all concentrations (10 µM: p < 0.001; 20 µM: p = 0.0002; 40 µM: p < 0.05), which suggests increasing apoptotic cell numbers. At 40 µM, there was also a higher percentage of cells arrested in G2/M compared to controls (p < 0.05, Figure 4(b)), indicating growth arrest. Concomitant VEGF levels also decreased (10 µM: 37%, p < 0.05; 20 µM: 18%, p < 0.0001; 40 µM: 28%, p < 0.0001, Figure 4(c)), another indication of cell inactivity or death.

786-O tumouroids which were cultured and treated at 1% O_2_ to mimic the tumour hypoxic environment appeared more drug resistant. Higher drug concentrations were needed to induce death in hypoxia with cells not responding to 10 µM Pazopanib, while there was significant death in normoxia for the same concentration. The pattern continued for higher concentrations with greater viability in hypoxia compared to normoxia (20 µM: 55.9% vs 43.6%; 40 µM: 65.7% vs 59.5%, NS) (Figure 4(d)).

Cell cycle analysis showed no difference between controls and 10 µM Pazopanib-treated, in agreement with CellTiter Glo. At higher concentrations, there was a significant increase in subG1 cells (20 µM: p < 0.001; 40 µM: p < 0.0001), an indicator of apoptotic cells (Figure 4(e)). VEGF levels were also significantly lower in treated tumouroids compared with controls (20 µM: 38.4%, p < 0.0001; 40 µM: 46.3%, p < 0.001), in agreement with viability and cell cycle results. In both normoxia and hypoxia, spheroid size agreed with viability, cell cycle and VEGF results, with a significant spheroid size decrease (p < 0.0001) in treated tumouroids versus controls (Figure 4(g)–(l)).

### Hypoxia decreases response to Pazopanib in CAKI-2 mature tumouroids

**Figure 5.**
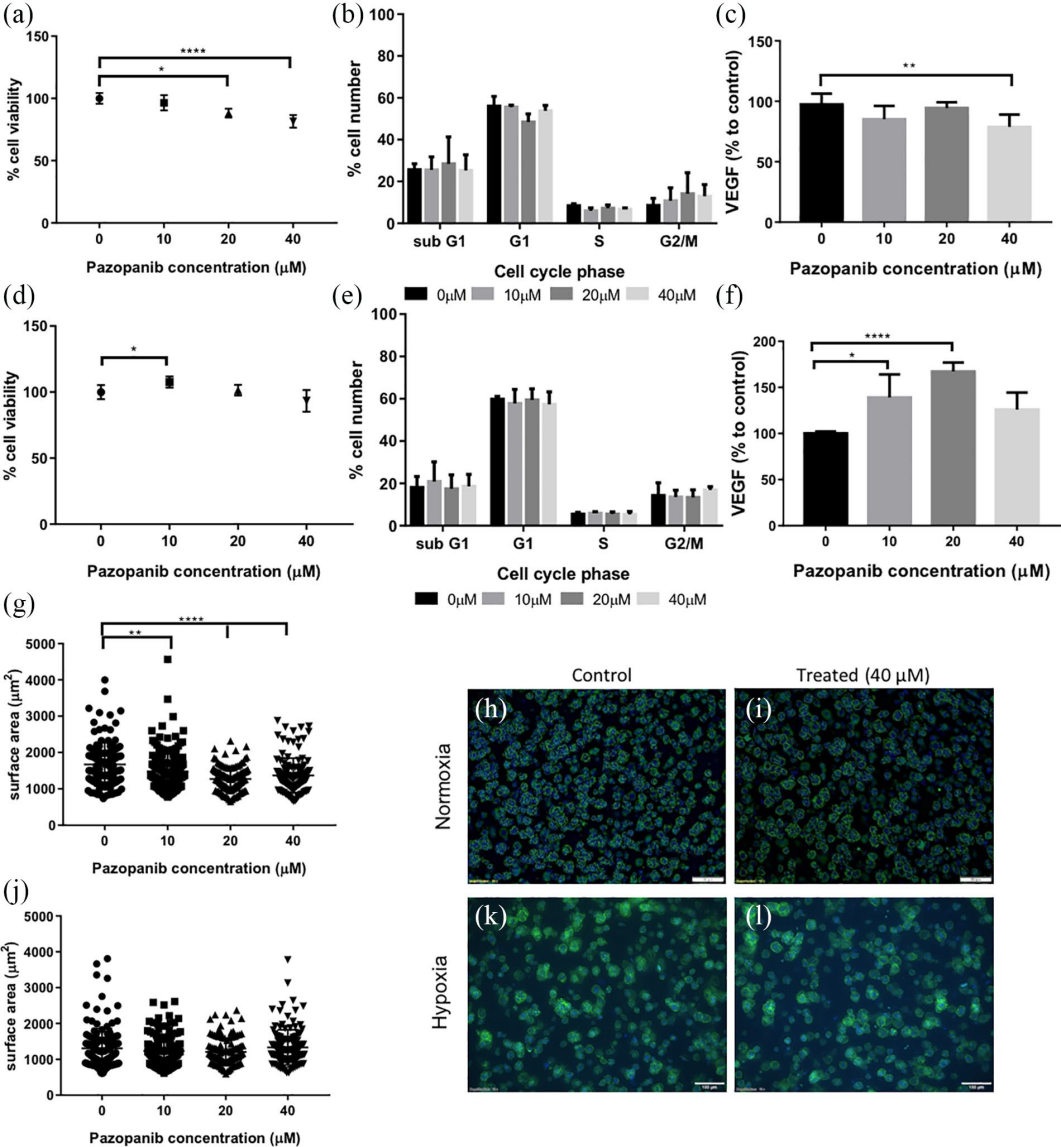
Mature tumouroids, manufactured using CAKI-2 cells, treated with Pazopanib. Tumouroids were exposed to Pazopanib for a total of 120 h on day 10 after manufacture, in normoxia or hypoxia. Normoxia (20% O_2_): a, b, c, g, h, i; hypoxia (1% O_2_): d, e, f, j, k, l. (a) Cell viability measured using CellTiter Glo. (b) Cell cycle analysis of treated tumouroids by flow cytometry. (c) VEGF levels in the supernatant of treated tumouroids measured using ELISA, shown as a percentage to untreated tumouroids. (d) Cell viability measured using CellTiter Glo treated with Pazopanib in hypoxia (1% O_2_). (e) Cell cycle analysis of treated tumouroids by flow cytometry. (d) VEGF levels in the supernatant of treated tumouroids shown as a percentage to untreated tumouroids. (g, j) Surface area of spheroids following treatment in (g) normoxia or (j) hypoxia demonstrating treatment effect on spheroid size. (h, i, k, l) Representative fluorescent images of cancer spheroids within mature tumouroids for (h, k) control and (i, l) treated tumouroids under (h, i) normoxia or (k, l) hypoxia (Phalloidin staining, DAPI counterstain; scale bar = 100 µm) *p < 0.05; **p < 0.01; ****p < 0.0001, Kruskal–Wallis with Dunn’s post hoc analysis.

In normoxia, CAKI-2 simple tumouroids were more resistant to Pazopanib than 786-O tumouroids, similar to the differential response in 2D. Although viability decreased significantly at 20 and 40 µM, it remained higher than in 786-O tumouroids, with 81.5% viability for 40 µM (Figure 5(a)). Cell cycle analysis showed no significant effect, while VEGF levels decreased ~20% at 40 µM (p < 0.01), similar to CellTiter Glo levels, but probably not sufficiently high for a meaningful biological effect at this drug concentration (Figure 5(b) and (c)).

CAKI-2 tumouroids cultured in hypoxia exhibited no significant decrease in viability or cell cycle analysis (Figure 5(d) and (e)). On the contrary, there was a significant increase in VEGF released in tumouroids treated at 10 and 20 µM compared with controls (10 µM: 139%, p < 0.05; 20 µM: 167%, p < 0.0001) (Figure 5(f)). In normoxia, spheroid sizes were significantly smaller with increasing Pazopanib concentrations (20 µM: p < 0.01; 40 µM: p < 0.0001) (Figure 5(g)–(i)), whereas in hypoxic conditions there was no effect on spheroid size (Figure 5(j)–(l)). Spheroids in normoxic simple CAKI-2 tumouroids were significantly smaller than those in 786-O tumouroids, at an average of 1674 versus 2470 µm^2^. CAKI-2 spheroids were significantly smaller in hypoxia (1305 µm^2^) rather than in normoxia (1674 µm^2^), suggesting hypoxia affects the development of 3D spheroids from single cells. VEGF levels were also lower in hypoxia, with VEGF in controls cultured in normoxia at ~4500 pg/mL compared with ~2400 pg/mL in hypoxia cultures.

### Pazopanib has detrimental effects against cancer and endothelial networks within complex tumouroids

**Figure 6.**
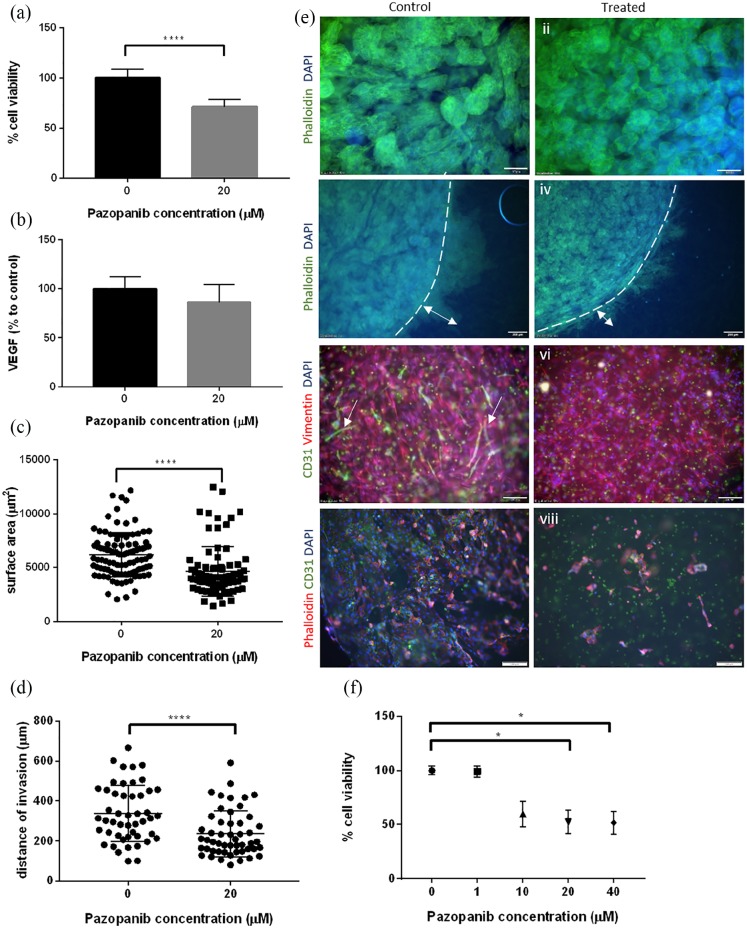
Pazopanib treatment of complex tumouroid manufactured using 786-O, HUVEC and HDF cells. (a) Cell viability (CellTiter Glo) following 7 days of treatment. (b) VEGF levels (% to control) in the supernatant following treatment measured by ELISA. (c) Surface area of spheroids within cancer mass in control and treated complex tumouroids. (d) Distance of invasion of 786-O cells within the stromal compartment in control and treated complex tumouroids. (e) Representative immunofluorescent images of control (left panel) and treated (right panel) cultures. (i–vi) Complex tumouroids and (vii–viii) HUVEC only; dotted line shows (iii, iv) cancer mass boundary and arrows indicate (iii, iv) 786-O invasion and (v) endothelial networks (scale bar = 100 µm in i, ii, v, vi, vii, viii; 200 µm in iii, iv). (f) Control cultures of HUVEC only, treatment on day 10 for 120 h. ****p < 0.0001; *p < 0.05 (a, b, c, d), Mann–Whitney U and (f) Kruskal–Wallis with Dunn’s post hoc analysis.

Complex tumouroids were manufactured using 786-O cells in the cancer compartment and HUVECs and HDFs in the stromal compartment. Based on results using 786-O simple tumouroids, a single drug concentration, 20 µM, was tested under normoxia. These conditions were chosen as they showed the greatest differential response to treatment in simple tumouroids and could be used to determine whether response on different cell populations would be measurable in a 3D multicellular model. Results showed a significant reduction (p < 0.0001) in viability in complex tumouroids, by 30% (Figure 6(a)). VEGF levels showed a decreasing trend, but no statistically significant difference (Figure 6(b)). This could be due to the presence of VEGF-producing HDFs or further paracrine loops between different cell populations. Imaging demonstrated a significant decrease in 786-O spheroid sizes (control: 6058 µm^2^, 20 µM: 3990 µm^2^, p <0.0001, Figure 6(c) and (e), i, ii), significantly decreased invasion into the surrounding stroma (control: 313.4 µm, 20 µM:195.6 µm, p < 0.0001, Figure 6(d) and (e), iii, iv) and a striking detrimental effect on endothelial viability and network formation (Figure 6(e), v, vi). It should be noted that spheroid sizes were larger in controls of complex tumouroids (6210 vs 2470 µm^2^) as culture time was increased by a further 2 days. While endothelial cells formed end-to-end networks in controls (length: 61–538 µm, mean: 161 µm) (Figure 6(e), v), there were no networks visible in treated tumouroids (Figure 6(e), vi). No drug effect was observed directly on HDFs, as the measurement was made difficult by the high number of vimentin-positive cells in both control and treated tumouroids (Figure 6(e), v, vi). The effects on HUVECs and 786-O cells are evident in mixed tumouroids as described; however, since ATP levels decreased by only 30% and VEGF levels were unaffected, we hypothesise that Pazopanib did not have a significant effect on HDFs in mixed tumouroids. Finally, we used HUVEC-only 3D cultures as controls since these cells are thought to be a main TKI target and showed a significant reduction in viability (50%, p < 0.05, Figure 6(f)), confirmed by morphology with few viable cells visible (Figure 6(e), vii, viii).

## Discussion

In vitro models are cheaper, give quicker results and are more ethical than animal models.^11^ However, in vitro drug testing does not necessarily give results that translate successfully, with only 10% candidate agents tested in clinical trials progressing to market use.^33^ The fact that anti-cancer agents, such as Pazopanib, do not necessarily kill cancer cells but attack the tumour microenvironment compounds the problem of what model to use.

We tested Pazopanib response in 2D cultures and 3D tumouroids made in dense extracellular matrix. We took a tissue-engineering approach, to enable precise incorporation of cellular and biophysical elements reflecting tumour conditions and demonstrated drug effects against RCC cells in addition to anti-angiogenic effects.

In 2D, Pazopanib killed 786-O and CAKI-2 RCC cells, with 786-O cells more responsive, at physiologically relevant doses circa IC50, 16 µM. The difference in response between the two cell lines could be related to their VHL status. As 786-O cells have a mutation in VHL, this leads to an increase in hypoxia-inducible factor (HIF) and downstream angiogenic genes such as VEGF.^1^ This could make cells more responsive to TKIs. There is contradictory evidence on TKI effects on RCC cells, for example, increased cell death of RCC lines treated with Sunitinib versus Pazopanib,^7^ and conversely, Sunitinib targeting endothelial, but not RCC, cells at physiological concentrations.^8^ A recent study by Roelants et al.^34^ tested Pazopanib effect on 786-O spheroids and showed no decrease in spheroid size. However, use of different cell lines (A498 and ACHN vs 786-O and CAKI-2) and different protocols (e.g. shorter treatment time by Roelants et al.^34^) makes comparisons between studies difficult.

The first biophysical characteristic we tissue-engineered in 3D was a dense cancer mass. We calculated matrix stiffness at ~5000 Pa (unpublished), closely resembling tumour tissue (4000 Pa), stiffer than Matrigel (180 Pa) and collagen hydrogels (330–1600 Pa), but softer than culture plastic (2.8 × 10^9^ Pa).^35,36^ 786-O cells showed significant Pazopanib-induced killing, regardless of the model used. Drug effects were confirmed using CellTiter Glo and imaging in all models, in addition to VEGF decrease and a shift to subG1 in cell cycle, indicative of apoptosis, in simple tumouroids. Unsurprisingly, drug exposure time had to be extended to demonstrate a strong killing effect in simple tumouroids versus 2D, for the same concentrations (10–40 µM). This reflects the biomimicry of drug penetration into a 3D mass rather than a cell sheet and has been described in other 3D models, for example, matrigel,^37^ collagen hydrogels, fibronectin^38^ and RCC spheres.^39^ In tumouroids, drug penetration is even more challenging as the cancer mass is surrounded by a dense 10% collagen matrix.^24^ Differential responses may also be linked to altered target receptor levels in 3D. Such changes were reported before^37,40,41^ including in our colorectal tumouroids where EGFR expression increased threefold in tumouroid-embedded cells versus 2D.^24^ The second line, CAKI-2, exhibited higher drug resistance both in 2D and in tumouroids. This was documented for CAKI-2 xenografts after sorafenib (TKI) treatment and could be related to the CAKI-2 VHL wild-type status.^42^

To increase biomimicry, we added a second biophysical parameter, hypoxia. The hypoxia pathway is important in ccRCC as VHL inactivation is found in ~90% of cases.^43^ In the absence of VHL, HIFα degradation is inhibited, leading to HIF1α accumulation and transcription of target angiogenic and proliferation genes, including VEGF.^44^ Our results concurred, with more VEGF produced by 786-O (VHL mutant) than CAKI-2 (wild-type) tumouroids. Both cell lines showed increased drug resistance in hypoxia compared to normoxia, in agreement with other in vitro studies, and could be related to changes in cell proliferation, protein or gene expression levels.^45,46^

We previously showed formation of end-to-end endothelial cell networks in complex tumouroids.^23,26,30^ Here, we assessed drug effect in different cell types. Treatment caused significant reduction in overall viability. Imaging confirmed decreased cancer invasion into the stroma^23,26^ alongside endothelial cell death and network disruption. We hypothesise that Pazopanib targeted primarily VEGFR1 and c-KIT for cancer cells and VEGFR2 for endothelial cells, based on reports which showed Pazopanib targeting VEGFR1 on multiple myeloma cells and VEGFR2 on HUVECs,^47^ and VEGFR1 and c-KIT on lung cancer cells and VEGFR2 on HUVECs.^48^ In mouse xenografts, Pazopanib reduced tumour volume and vasculature, while in vitro experiments suggested its action via VEGFR2 (endothelial cells) and PDGFRβ (fibroblasts).^49^ HUVEC and HDF incorporation may not capture the full complexity of the cancer stroma; however, to our knowledge, this is the first study assessing drug response in the tumour microenvironment in a 3D in vitro renal cancer model.

In the era of personalised medicine, there has been a shift from using cell lines towards patient-derived cells as more relevant.^10,15^ Differences in the treatment response of the two cell lines used highlight the importance of considering RCC subtype and inter-patient heterogeneity when choosing therapeutic interventions. Developing an easy-to-use medium throughput pipeline suitable for drug testing in cell lines and patient-derived cells could revolutionise pre-clinical research and be of great use to the pharmaceutical industry. We propose that tumouroids, which are reproducible, versatile and easy to analyse, are highly suited to this purpose.

We are currently manufacturing tumouroids with patient-derived cells and testing Pazopanib responses as a predictive tool for personalised patient care.^28^ Our future work aims to further increase model complexity, incorporating an immune component, to test immunotherapies. Immune-competent models have also been created by others (reviewed by Nyga et al.^50^ with different approaches and cell populations used). Our approach will incorporate lymphocytes and antigen-presenting cells isolated from peripheral blood to test effects of drugs used for treating RCC, such as Nivolumab or Ipilimumab. This will make tumouroids more applicable to the ever increasing landscape of available treatments.^1,51,52^

## Conclusion

In this study, we describe an in vitro 3D model which can be used to evaluate drugs which target both the cancer mass and the angiogenic component of the model. We tissue-engineered renal tumouroids which mimic the multicellularity and dense nature of cancer tissues. We demonstrated response to Pazopanib, which significantly reduced overall viability and cancer invasion, and disrupted endothelial networks. Tumouroids can be adapted for use with other cancer lines or patient-derived cells, and by increasing complexity, they can be used for testing immunotherapies and other novel agents.

## Supplemental Material

Supplementary_figures_Stamati_et_al – Supplemental material for The anti-angiogenic tyrosine kinase inhibitor Pazopanib kills cancer cells and disrupts endothelial networks in biomimetic three-dimensional renal tumouroids Click here for additional data file. Supplemental material, Supplementary_figures_Stamati_et_al for The anti-angiogenic tyrosine kinase inhibitor Pazopanib kills cancer cells and disrupts endothelial networks in biomimetic three-dimensional renal tumouroids by Katerina Stamati, Patricia A Redondo, Agata Nyga, Joana B Neves, Maxine GB Tran, Mark Emberton, Umber Cheema and Marilena Loizidou in Journal of Tissue Engineering
