# Supplementary material for: The anti-angiogenic tyrosine kinase inhibitor Pazopanib kills cancer cells and disrupts endothelial networks in biomimetic three-dimensional renal tumouroids
Source: J Tissue Eng. 2020 May 18;11:2041731420920597. doi: 10.1177/2041731420920597 (PMC7238304; doi:10.1177/2041731420920597)
Supplement: Supplementary_figures_Stamati_et_al – Supplemental material for The anti-angiogenic tyrosine kinase inhibitor Pazopanib kills cancer cells and disrupts endothelial networks in biomimetic three-dimensional renal tumouroids [file Supplementary_figures_Stamati_et_al.pdf]

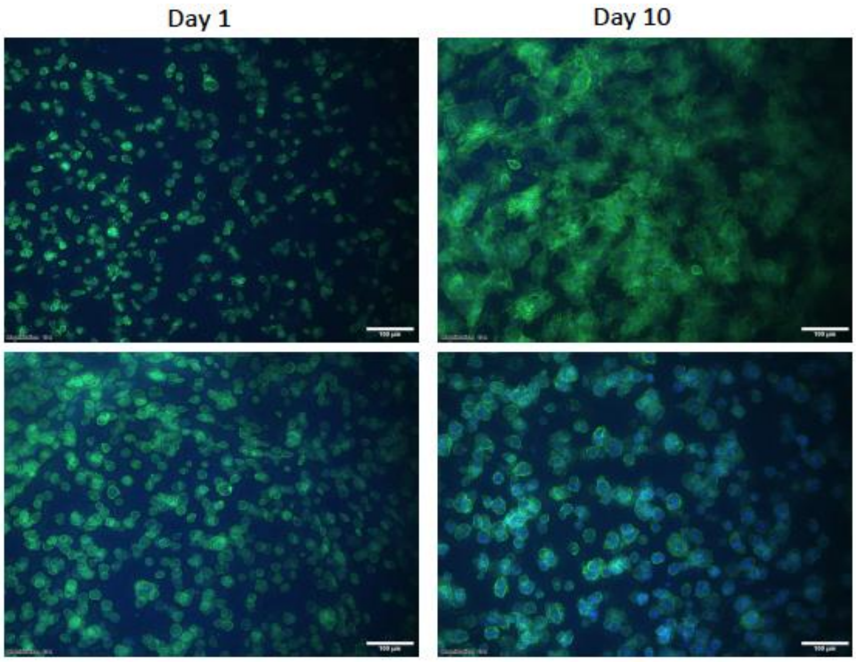

Supplementary figure 1: Tumouroids on day 1 and day 10 of culture in 786-O (upper panel) and CAKI-2 lower panel. On day 1 tumouroids have single cells and on day 10 cells have formed spheroids/ cell aggregates. Phalloidin staining, DAPI counterstain, scale bar= 100µm

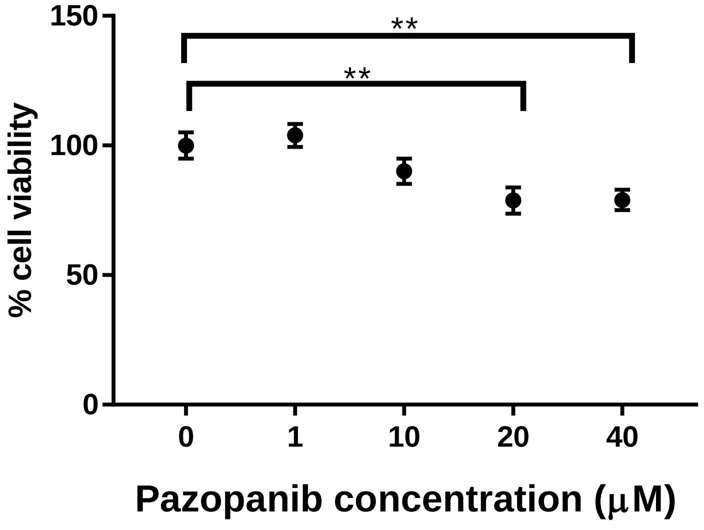

Supplementary figure 2: 786-O simple tumouroids treated on day 1 for 48hours with Pazopanib.

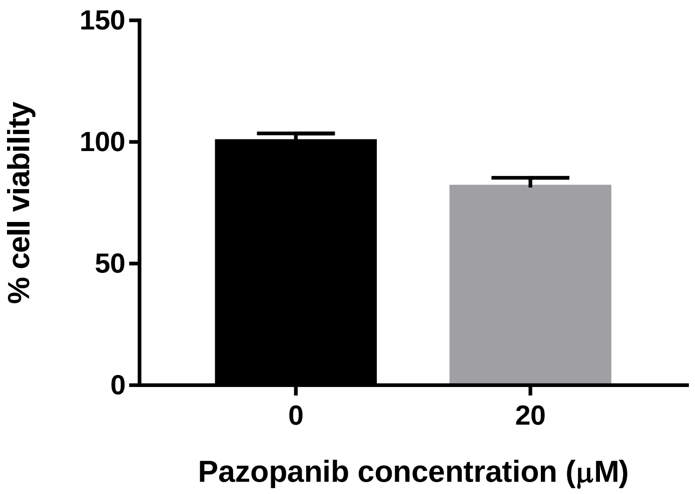

Supplementary figure 3: Complex tumouroids with 786-O, HUVEC and HDF cells treated for 120 hours with Pazopanib. No significant decrease in cell viability was observed.

Control Treated

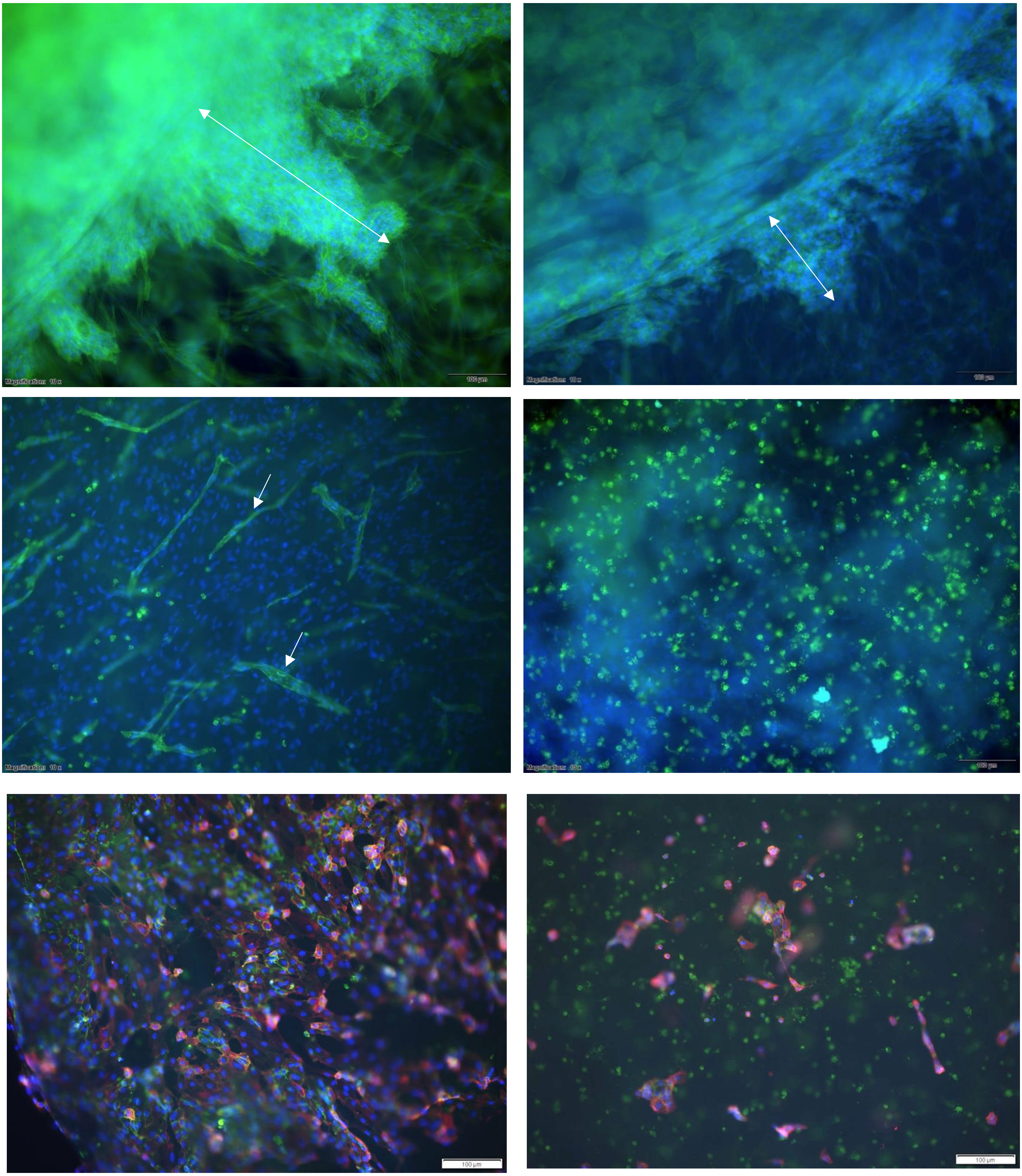

Supplementary figure 4: Immunofluorescence images of complex tumouroids, controls and treated. A, B) Invasion of 786-O cells into the stroma C,D) endothelial cell networks , E,F) Endothelial cells in HUVEC only control cultures
